# A Novel PET Imaging Probe for the Detection and Monitoring of Translocator Protein 18 kDa Expression in Pathological Disorders

**DOI:** 10.1038/srep20422

**Published:** 2016-02-08

**Authors:** Mara Perrone, Byung Seok Moon, Hyun Soo Park, Valentino Laquintana, Jae Ho Jung, Annalisa Cutrignelli, Angela Lopedota, Massimo Franco, Sang Eun Kim, Byung Chul Lee, Nunzio Denora

**Affiliations:** 1 Department of Pharmacy – Drug Sciences, University of Bari “A. Moro”, Bari, Italy; 2 Department of Nuclear Medicine, Seoul National University College of Medicine, Seoul National University Bundang Hospital, Seongnam, Republic of Korea; 3 Department of Transdisciplinary Studies, Graduate School of Convergence Science and Technology, Seoul National University, Seoul, Republic of Korea; 4 Center for Nanomolecular Imaging and Innovative Drug Development, Advanced Institutes of Convergence Technology, Suwon, Republic of Korea

## Abstract

A new fluorine-substituted ligand, compound **1** (CB251), with a very high affinity (Ki = 0.27 ± 0.09 nM) and selectivity for the 18-kDa translocator protein (TSPO), is presented as an attractive biomarker for the diagnosis of neuroinflammation, neurodegeneration and tumour progression. To test compound **1** as a TSPO PET imaging agent *in vivo*, 2-(2-(4-(2-[^18^F]fluoroethoxy)phenyl)-6,8-dichloroimidazo[1,2-a]pyridin-3-yl)-*N,N*-dipropylacetamide ([^18^F]**1**; [^18^F]CB251) was synthesized by nucleophilic aliphatic substitution in a single-step radiolabelling procedure with a 11.1 ± 3.5% (*n* = 14, decay corrected) radiochemical yield and over 99% radiochemical purity. In animal PET imaging studies, [^18^F]CB251 provided a clearly visible image of the inflammatory lesion with the binding potential of the specifically bound radioligand relative to the non-displaceable radioligand in tissue (BP_ND_ 1.83 ± 0.18), in a neuroinflammation rat model based on the unilateral stereotaxic injection of lipopolysaccharide (LPS), comparable to that of [^11^C]PBR28 (BP_ND_ 1.55 ± 0.41). [^18^F]CB251 showed moderate tumour uptake (1.96 ± 0.11%ID/g at 1 h post injection) in human glioblastoma U87-MG xenografts. These results suggest that [^18^F]CB251 is a promising TSPO PET imaging agent for neuroinflammation and TSPO-rich cancers.

Microglia constitute up to 10% of the total cell population of the brain and make up the largest resident population of macrophages, which play an important role in the pathogenesis of several neurodegenerative disorders. In fact, in response to a wide variety of central nervous system (CNS) insults, microglia change their morphological state from a resting phenotype to an activated phenotype^1^. To date, various degrees of microglia activation have been observed in neurodegenerative disorders. Relevant cases include the high density of microglia surrounding the amyloid plaques, which are a hallmark pathology in Alzheimer´s disease (AD) and the abundant presence of activated microglia in zones of demyelination in multiple sclerosis^2^,^3^. Although it is well accepted that microglia activation contributes to the pathogenesis of several neurodegenerative diseases, including AD, multiple sclerosis and HIV-associated dementia, to date its detection is predominantly restricted to histopathological techniques utilizing post-mortem CNS tissues^1^. Computerized tomography and magnetic resonance imaging technologies allow morphometric documentation of late-stage changes in brain volume but are generally not regarded as sensitive or early detectors of brain damage. Recent studies have suggested that activated microglia in the CNS may be detected *in vivo* using positron emission tomography (PET) with the aid of pharmacological ligands of the mitochondrial translocator protein 18 kDa (TSPO)^4^. Furthermore, TSPO levels appear to be enhanced in cancer cells, including brain, breast, colon, prostate and ovarian cancers, as well as in astrocytomas and hepatocellular and endometrial carcinomas^5^,^6^.

Therefore, the successful development of a TSPO-specific PET tracer may help to chart the progression of pathologies in which TSPO is overexpressed, as well as to assess the efficacy of therapies designed to control these diseases. Potent ligands for TSPO have been identified from many different structural classes, such as isoquinoline carboxamides (e.g., PK 11195)^7^, benzodiazepines (e.g., Ro-54864)^8^, phenoxyarylacetamides (e.g., DAA1106)^9^, aryloxyanilides (e.g., PBR28)^10^ and 2-phenyl-imidazo[1,2-a]pyridine acetamides (e.g., alpidem)^11^. Many of these ligands have been developed to visualize activated microglia via several imaging techniques. In this context, we recently proposed a practical and easy approach to achieve this objective using fluorescent probes chemically linked to 2-phenyl-imidazo[1,2-a]pyridine acetamide TSPO ligands^12^,^13^. It should also be mentioned that the first examples of targeted nanocarriers for imaging TSPO *in vitro* using fluorescent probes have recently been presented^14^,^15^. Regarding PET, potent TSPO ligands have been explored as potential radioligands. Most of these are carbon-11-radiolabelled TSPO ligands such as the well-known [^11^C]PK 11195, which has been used to image TSPO in pathological states such as neuroinflammatory diseases and tumours by either autoradiographic methods or PET^16^. Although these studies established the potential to image TSPO in pathologies that overexpress this mitochondrial protein, they also highlighted important limitations of [^11^C]PK 11195 as a molecular imaging probe, such as a high level of nonspecific binding, a low signal-to-noise ratio and nonspecific uptake^17^. Therefore, a number of alternative TSPO PET radioligands for neuroinflammatory imaging have been developed over the last several years. We recently reported the development of novel imidazopyridine acetamide PET ligands with improved *in vivo* specificity for TSPO^18^,^19^. In particular, distribution studies in mice showed that the most promising *N*-[^11^C]methylated imidazopyridine acetamide accumulated in TSPO-rich regions and that the radioactivity in brain regions was reduced by co-injection of PK 11195^18^. Nevertheless, the use of such radioligands is necessarily restricted to imaging facilities either at or very close to their site of production because of the short half-life of carbon-11 (20.38 min). The use of fluorine-18 (half-life 109.7 min) as an alternative label offers several advantages over carbon-11. First, high (multi-Ci) activities of fluorine-18, such as [^18^F]fluoride, can be produced from moderate energy cyclotrons by the ^18^O(p,n) ^18^F reaction on [^18^O] water. Second, the longer half-life allows transport over considerable distances to remote PET centres and also allows longer and more accurate imaging sessions. Finally, fluorine-18 decays by emitting a low-energy positron, which permits the high spatial resolution of modern PET cameras to be fully utilized.

To date, only a few ^18^F-labelled TSPO ligands have been reported. One of these is a close analogue of [^11^C]PK 11195, namely, 1-(2-[^18^F]fluoro-5-nitrophenyl)-*N*-methyl-*N*-(1-methylpropyl)-3-isoquinoline carboxamide ([^18^F]PK 14105), which was shown to be a marker of brain ischaemic lesions in rats but has not yet been evaluated in humans^20^. Another such ligand is the recently reported high-affinity [^18^F]fluoropropyl analogue of iodo-PK 11195, whose imaging properties have not yet been described^21^. However, even the most promising of these radioligands suffers from extensive metabolism and defluorination. In the last few years, considerable effort has gone into the development of new radioligands that target TSPO. Thus, radioligands from imidazopyridine and other structural classes with improved *in vivo* specificity for TSPO and with the potential to improve PET imaging of TSPO expression have been synthesized and evaluated in animals^22^,^23^. Recently, the first examples of ^18^F-labelled alpidem analogues, also known as [^18^F]PBR102 and [^18^F]PBR111, have been assessed *in vivo* using PET^23^,^24^. Alpidem has been shown to act on both TSPO and central benzodiazepine receptors (CBRs), with a preference towards TSPO. In an effort to increase this selectivity, some groups have synthesized a series of potent, imidazo[1,2-a]pyridine-based TSPO ligands by introducing various substituents onto the alpidem structure at positions 6 and 8 of the imidazo[1,2-a]pyridine nucleus (Fig. 1). In particular, the main results of our previous structure-activity-relationship studies, combined with a pharmacophore model of TSPO ligands, revealed that substituents at the 8-position (Y, Fig. 1), such as a chlorine atom, lead to compounds with enhanced TSPO selectivity (K_i_(CBR)/K_i_(TSPO)>10^4^)^11^. To our knowledge, no ^18^F-labelled ligands have been developed from the 6,8-di-substituted imidazo[1,2-a]pyridin-*N*,*N*-dipropylacetamides class and evaluated by PET imaging in animal or human subjects. Through our efforts to develop new TSPO ligands, we now report the synthesis and *in vitro* and *in vivo* evaluation of the novel ^18^F-labelled 2-phenyl-imidazo[1,2-a]pyridine analogue [^18^F]**1**, named [^18^F]CB251 in Fig. 1, as a TSPO-selective PET radiotracer that is useful for imaging activated microglia, as well as TSPO-rich brain tumours.

## Results

### Synthesis of CB251 and the tosylate precursor

**Figure 2:**
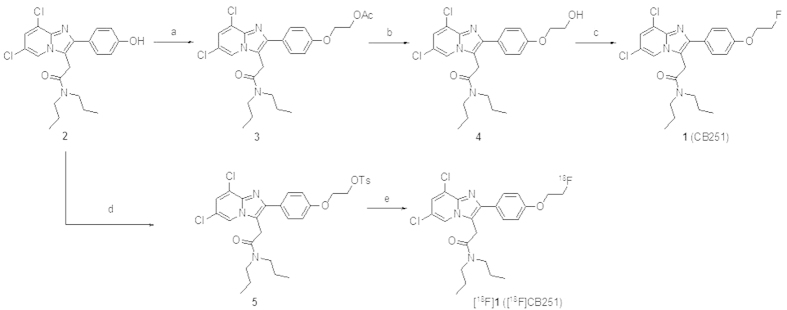
Schematic synthesis of CB251 and [^18^F]CB251. Reagents and conditions: (**a**) Ethyl 2-bromoacetate, K_2_CO_3_, KI, *n*Bu_4_NI, anhydrous DMF, r.t., overnight; (**b**) CsCO_3_, CH_3_OH:H_2_O = 2:1, r.t., overnight; (**c**) DAST, anhydrous CH_2_Cl_2_, r.t., 3 h; (**d**) NaH, 1,2-di(tosyloxy)ethane, anhydrous THF, r.t., overnight; and (**e**) *n*Bu_4_N[^18^F]F complex, *t*-BuOH, 120 °C, 10 min.

To synthesize authentic compound **1** (CB251), 2-(6,8-dichloro-2-(4-hydroxyphenyl)imidazo[1,2-a]pyridin-3-yl)-*N*,*N*-dipropylacetamide (**2**) was prepared according to synthetic procedures reported elsewhere^11^. In brief, as illustrated in Fig. 2, ethyl-acetate-substituted compound **3** was synthesized via alkylation of the phenolic compound **2** in the presence of ethyl 2-bromoacetate, K_2_CO_3_, KI and *n*Bu_4_NI in anhydrous DMF. The acetyl moiety of **3** was hydrolysed using CsCO_3_ in a CH_3_OH:H_2_O (2:1) solution, leading to 2-(2-(4-(2-hydroxyethoxy)phenyl)-6,8-dichloroimidazo[1,2-a]pyridin-3-yl)-*N,N*-dipropylacetamide (**4**). CB251 was easily obtained by fluorination of compound **4** using a solution of DAST in anhydrous CH_2_Cl_2_. The tosylate precursor (**5**) for the radiofluorination was prepared from **2** using NaH and di(tosyloxy)ethane in anhydrous THF.

### Radiosynthesis

The radiosynthesis of [^18^F]CB251 was accomplished starting from the tosylate precursor **5** via nucleophilic aliphatic substitution under various reaction conditions. Table 1 summarizes the results of the [^18^F]fluorine incorporation yields that were achieved using different bases, solvents, reaction times and temperatures. Among the reaction conditions, promising results were obtained when *n*Bu_4_NHCO_3_ (4.0 equiv. relative to the precursor) and compound **5** were used in the presence of *tert*-butanol as the reaction solvent at 120 °C for 10 min (Table 1, entry 7). The reaction mixture was separated by via semi-preparative HPLC and the product was collected after approximately 20 min (Fig. S1). Using the optimized conditions described above, the radiochemical yield of the final formulated [^18^F]**1** product was 11.1 ± 3.5% (*n* = 14, decay corrected) within a synthesis time of 60 min, including HPLC purification. The specific activity of [^18^F]**1** at the end of the synthesis ranged from 104 to 154 GBq/μmol. The identity was confirmed by co-injection with authentic compound using analytical HPLC (Fig. S2).

### Log D determination and stability of [^18^F]CB251 in human serum

The ability of [^18^F]CB251 to cross the blood-brain barrier (BBB) by passive diffusion was assessed by measuring the partition coefficient (log D), whose experimental value was 3.00 ± 0.03. This result suggested that [^18^F]CB251 can readily cross the BBB and indicated a somewhat high lipophilicity compared with [^11^C]PBR28 (log D = 2.82 ± 0.03)^22^. In addition, the *in vitro* stability of [^18^F]CB251 in human serum demonstrated that greater than 99% of the radioactive parent remained after 2 h (Fig. S3).

### *In vitro* binding affinity

CB251 was next assayed for its binding affinity to TSPO and CBR by measuring its ability to displace [^3^H]PK 11195 and [^3^H]flunitrazepam, respectively^24^. As shown in Table 2, the affinities for TSPO and CBR, expressed as inhibition constants (K_i_), were compared with those of unlabelled PK 11195, PBR28 and flunitrazepam. CB251 is characterized by a subnanomolar binding affinity for TSPO (K_i_ = 0.27 ± 0.09 nM), which was 5 and 22 times higher than that observed for PK 11195 (K_i_ = 1.38 ± 0.42 nM) and PBR28 (K_i_ = 6.1 ± 6.4 nM)^25^, respectively. Furthermore, binding to CBR was below the detection limit, indicating the high selectivity of CB251 for TSPO.

### *Ex vivo* tissue distribution

Purified [^18^F]CB251 was reconstituted in a 10% ethanol-saline solution. Animals were killed at various times after tail-vein injection of [^18^F]CB251 (0.74 MBq/0.2 mL per mouse) and the radioactivity concentrations were measured in various tissues upon dissection. The results of the tissue biodistribution experiment are shown in Table 3. [^18^F]CB251 accumulated strongly in TSPO-enriched tissues such as the lung, heart and kidney, whereas it exhibited comparatively low uptake in tissues such as the liver and stomach. Additionally, the spleen and adrenal glands, which are particularly rich in TSPO^26^, exhibited high radioactivity (25.11 ± 2.16 and 22.60 ± 5.00%ID/g at 15 min post injection). [^18^F]CB251 also showed faster blood clearance at early time points (4.39 ± 0.78%ID/g at 5 min and 0.60 ± 0.12%ID/g at 60 min post injection). The brain uptake of [^18^F]CB251 at 5 min post injection was 2.89 ± 0.23%ID/g, followed by a gradual reduction to significantly lower levels (0.53 ± 0.23%ID/g at 120 min post injection). The radioactivity concentration in the femur remained low for 120 min (1.19 ± 0.50%ID/g at 5 min post injection; 1.49 ± 0.80%ID/g at 120 min post injection).

### PET study in LPS-induced neuroinflammation and tumour models

**Figure 3:**
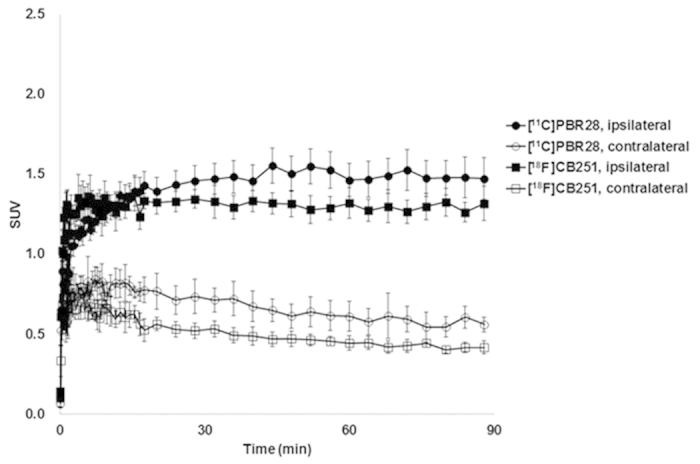
Time-activity curve of [^18^F]CB251 and [^11^C]PBR28 in an LPS-induced neuroinflammation rat model (n = 5).

**Figure 4:**
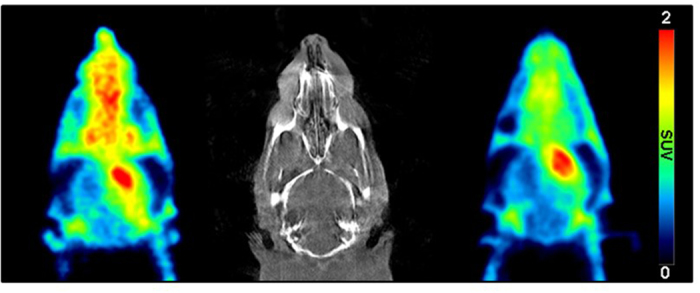
Head CT image of an LPS-induced neuroinflammation rat model (middle). PET images of the same animal receiving [^11^C]PBR28 (left) and [^18^F]CB251 (right). The PET images shown are averaged images from 0 to 90 min after injection.

**Figure 5:**
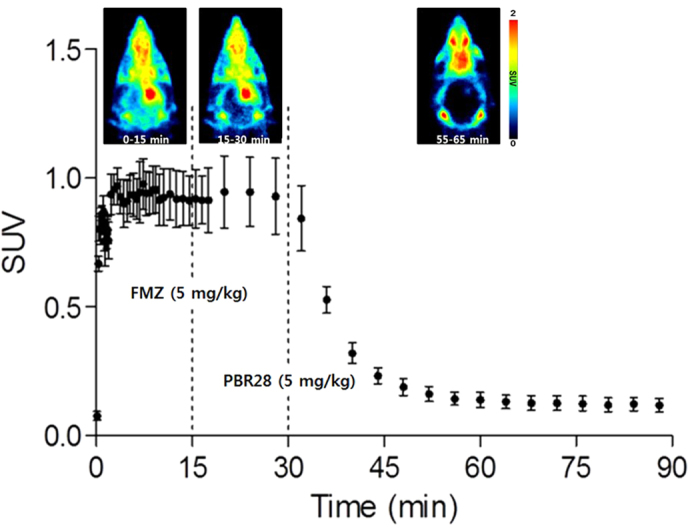
Representative axial PET images and time-activity curves of [^18^F]CB251 in an LPS-induced neuroinflammation rat model before and after flumazenil and PBR28 displacement.

To further examine the potential of [^18^F]CB251, it was compared directly to [^11^C]PBR28 in a rat lipopolysaccharide (LPS)-induced neuroinflammation model using sequential microPET scans of [^11^C]PBR28 and [^18^F]CB251 (Figs 3 and 4). Figure 3 shows the time-courses of [^18^F]CB251 and [^11^C]PBR28 distribution in the ipsilateral (right) and contralateral (left) striatum of the rat brain. Both radioligands had similar pharmacokinetic profiles, but the uptake of [^11^C]PBR28 in the ipsilateral region was slightly higher than that of [^18^F]CB251 during the PET scan time. Summed microPET images showed significant [^18^F]CB251 binding in the ipsilateral striatum in the same inflamed rat brain (Fig. 4). Additionally, there was no significant uptake in the skull. In the blocking experiment with consecutive injection of flumazenil and PBR28, flumazenil did not prevent the uptake of [^18^F]CB251, whereas uptake in the ipsilateral area decreased significantly after PBR28 injection (Fig. 5).

**Figure 6:**
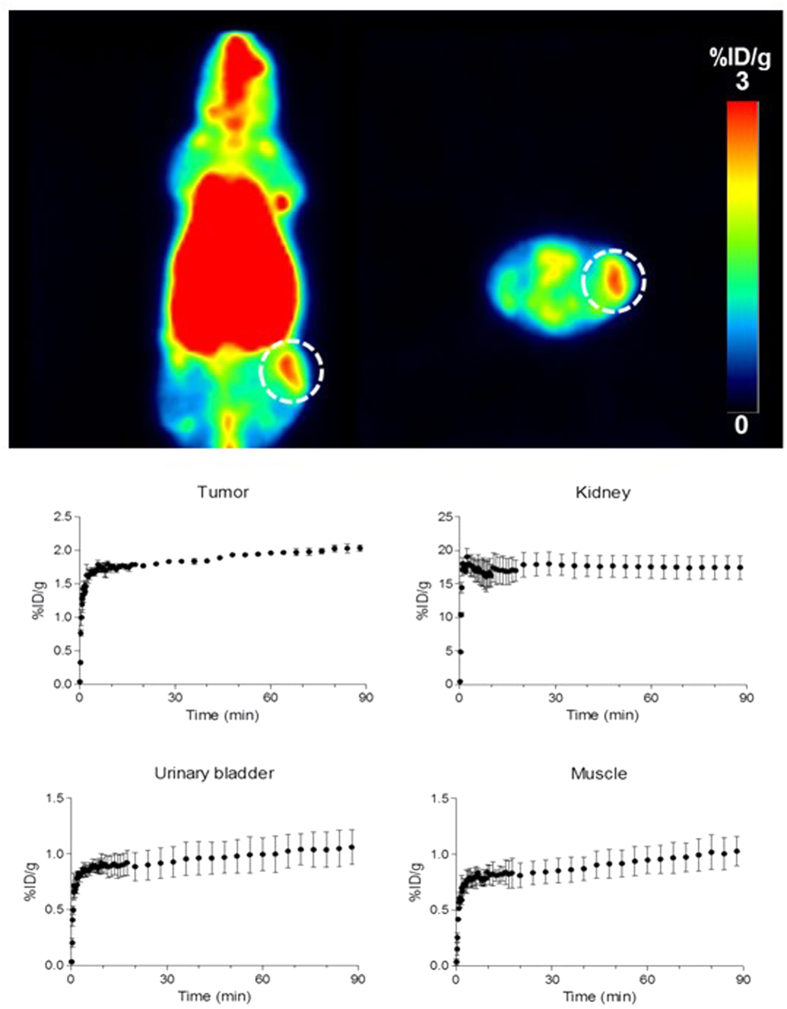
Representative coronal and axial PET images summed between 40 to 60 min of [^18^F]CB251 in a U87-MG tumour-bearing nude mouse (upper) and *in vivo* biodistribution of [^18^F]CB251 in tumour, kidney, bladder and muscle using PET image ROI-derived radiouptake percentage of injected dose per gram (bottom). The results represent the mean ± SD (n = 3). The tumour is denoted by the dotted circle.

Another *in vivo* test of [^18^F]CB251 was performed in U87-MG-bearing mice using a microPET scanner (Fig. 6). Tumour cells were engrafted on the left thigh of Balb/c nude mice. [^18^F]CB251 was administered intravenously after the tumour volume reached approximately 364.5 ± 32.2 mm^3^ and a dynamic PET scan was performed. [^18^F]CB251 showed high kidney uptake, as well as low bladder and muscle uptake. In particular, [^18^F]CB251 provided a tumour accumulation (1.96 ± 0.11%ID/g at 1 h post injection) and a visible image of the tumour in summed PET images.

### Immunohistochemical study

**Figure 7:**
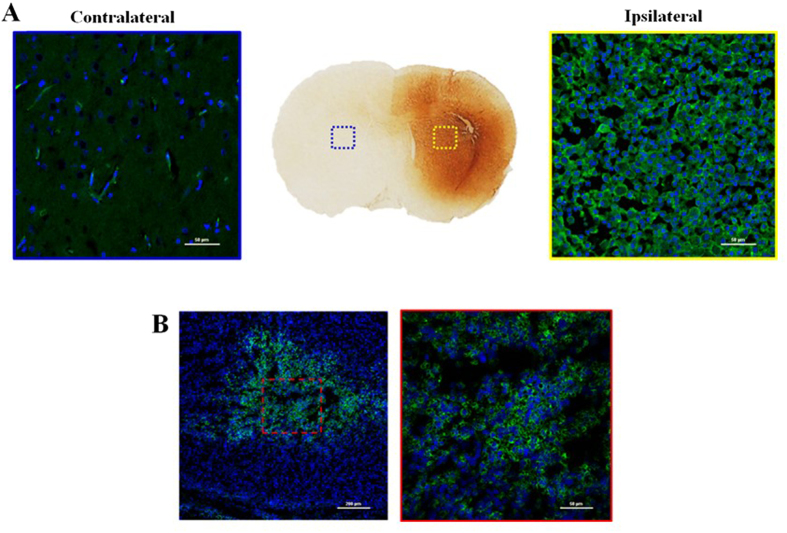
The immunohistochemical staining (anti-CD68; Serotec, Oxford, UK) of the ipsilateral (left: 200x) and contralateral areas (right: 200x) in LPS-induced inflammation rat brain sections (**A**) and human glioblastoma U87-MG tumour tissue (left: 100x, right: 400x) (**B**). Blue colour: DAPI, green colour: anti-CD68-FITC.

Histological examinations of dissected brain and tumour tissues were performed in all of the LPS-induced neuroinflammation rats and U87-MG-bearing mice used for the microPET scans to investigate microglial activation by CD68 staining. The selective PET neuroinflammation images of [^18^F]CB251 in the ipsilateral area showed a high density of CD68-positive activated microglia compared with the contralateral area (Fig. 7A). In addition, treatment with a CD68 antibody strongly revealed positive immunostaining inside the human glioblastoma U87-MG tumour (Fig. 7B).

## Discussion

The aim of this study was to synthesize and evaluate a fluorine-18 labelled analogue of alpidem, namely, [^18^F]CB251, for *in vivo* imaging of TSPO in both neuroinflammation and TSPO-rich tumour models. Our initial strategy was to synthesize various potent and selective PBR ligands mainly characterized by various substituents at 6- and 8-positions of the imidazo[1,2-a]pyridine nucleus and several molecules in spired by the novel imidazopyridine acetamides such as *N*-[^11^C]methylated imidazopyridine acetamides ([^11^C]CB148), [^11^C]CB184 and [^11^C]CB190 with improved *in vivo* specificity for TSPO were investigated^11^,^18^,^19^. As an extension of our efforts to develop new TSPO-selective PET radiotracers, we have generated the first ^18^F-labelled ligand, [^18^F]CB251, from the 6,8-di-substituted imidazo[1,2-a]pyridin-*N*,*N*-dipropylacetamide class and evaluated its performance *in vivo*. The first step of this study was the synthesis of CB251, which was accomplished with good yields following the well-known synthetic procedures outlined in Fig. 2. As predicted, CB251, which is characterized by the presence of a chlorine atom at the 8-position of the imidazo[1,2-a]pyridine nucleus, displays high affinity and selectivity towards TSPO (K_i_ = 0.27 ± 0.09 nM).

Fluorine-18 labelling of CB251 was performed by nucleophilic aliphatic substitution of the corresponding tosylate **5** (precursor for labelling) using [^18^F]fluoride (Fig. 2). Various conditions were explored for the preparation of the TSPO ligand [^18^F]CB251. Above all, when the precursor **5** for labelling was reacted with *n*Bu_4_N[^18^F]F in aprotic solvents (i.e., CH_3_CN, DMSO or DMF) under general anhydrous radiofluorination conditions, the yield of the desired product was somewhat low (Table 1, entries 1-3). In fact, after radiofluorination, the amount of unreacted precursor **5** was over 90% according to TLC. Given that these low radiochemical yields might be due to the use of aprotic polar solvents, the use of *tert*-butanol as the reaction solvent increased the yield (Table 1, entry 4) and extending the reaction time did not improve the reaction yield (Table 1, entry 5). In contrast, increasing the concentration of *n*Bu_4_NHCO_3_ from 0.8 to 2.0 equivalents relative to the precursor **5** positively affected the radiochemical yield (Table 1, entry 6). By changing the base from *n*Bu_4_NHCO_3_ to K_2.2.2_/K_2_CO_3_, a different pattern was observed in our experiment. Indeed, even though a reasonable radiochemical yield was achieved using K_2.2.2_/K_2_CO_3_ (Table 1, entry 9) as the base, suddenly increasing the equivalents of K_2_CO_3_ reduced the reaction yield (Table 1, entry 10). Consequently, [^18^F]CB251 was synthesized in the desired yields by employing a *tert*-butanol solvent, namely, 4.0 equivalents of *n*Bu_4_NHCO_3_ relative to the precursor **5**, at 120 °C for 10 min (Table 1, entry 7). After pre-purification on a Sep-Pak cartridge and purification via HPLC, [^18^F]CB251 was formulated and ready for use, with a 10% EtOH/saline solution and an 11.1 ± 3.5% radiochemical yield (decay corrected for 60 min). In addition, the measured log D value, which predicted good *in vitro* BBB permeability, together with the high stability in human serum indicated that [^18^F]CB251 was sufficiently stable for further use *in vivo*.

Tissue distribution studies in healthy mice confirmed that [^18^F]CB251 had a significantly high uptake (>22%ID/g) in TSPO-enriched tissues, as well as a rapid blood clearance. In the brain, which is the principal target tissue for neuroinflammation, the initial uptake of [^18^F]CB251 showed a desirable level of 2.89 ± 0.23%ID/g and its radioactivity was rapidly washed out from the brain at 30 and 60 min post injection (1.39 ± 0.13 and 0.63 ± 0.24%ID/g, respectively). However, the low uptake of radioactivity in the femur (<2%ID/g) indicated negligible defluorination of the radioligand and reflected the high *in vitro* stability recorded for [^18^F]CB251 in human serum. Therefore, [^18^F]CB251 was expected to be sufficiently stable for successive *in vivo* PET imaging studies.

In microPET imaging studies, the accuracies of [^18^F]CB251 and [^11^C]PBR28 were determined by comparing the imaging data. Although [^18^F]CB251 showed a slightly lower uptake in the ipsilateral region compared with [^11^C]PBR28, it rapidly approached the highest target-to-background ratio at early imaging times and selectively accumulated in the ipsilateral striatum. As a consequence, the uptake ratio of [^18^F]CB251 between the ipsilateral area and the contralateral area was similar to that of [^11^C]PBR28 at 30 min post injection (2.35 times for [^18^F]CB251 and 2.25 times for [^11^C]PBR28). In addition, [^18^F]CB251 rapidly reached a steady state in the ipsilateral area, as well as in the contralateral, area compared with [^11^C]PBR28. These results indicate that [^18^F]CB251 stained the inflammatory lesion with the highest target-to-background ratio at early imaging times. Representative PET images showing the differences in radiotracer accumulation in the brains of LPS-induced neuroinflammation rats are shown in Fig. 4.

To confirm the TSPO selectivity and specificity of [^18^F]CB251, displacement studies were performed in the LPS-induced inflammation rat model by injecting an excess of flumazenil at 15 min post [^18^F]CB251 injection, which is the time at which the [^18^F]CB251 uptake displays a steady state in the ipsilateral region. Flumazenil, which selectively binds to CBR, did not prevent the uptake of [^18^F]CB251 in the ipsilateral region. However, uptake in the ipsilateral area was significantly decreased following PBR28 injection. These displacement studies indicate that the uptake of the major fraction of [^18^F]CB251 in the inflammatory lesion was selectively and specifically mediated by TSPO, as shown in Fig. 5.

In the TSPO-rich cancer imaging study, the PET image of [^18^F]CB251 visually reflected the *ex vivo* biodistribution of ^18^F uptake in mice. In fact, [^18^F]CB251 showed high uptake in TSPO-rich organs after injection, including the lung, heart and kidneys and only minor radioactivity was excreted in the urine (Fig. 6). The high kidney uptake and slow renal clearance might be explained by the strong TSPO binding affinity of [^18^F]CB251 in these organs, which are characterized by high TSPO expression levels. The pronounced liver uptake is probably correlated with the lipophilic nature of CB251. In particular, [^18^F]CB251 displayed reasonable tumour uptake in the human glioblastoma U87-MG xenograft model (1.96 ± 0.11%ID/g at 1 h post injection) and was comparable to accumulation of other TSPO ligands^27^. TSPO expression in tumour sections as well as in LPS-induced inflammation brain sections was determined by CD68 immunohistochemical staining (Fig. 7). In particular, the PET neuroinflammation images of [^18^F]CB251 in the ipsilateral region showed a high density of CD68-positive activated microglia compared with the contralateral area (Fig. 7A). Furthermore, treatment with a CD68 antibody revealed strong positive immunostaining inside the human glioblastoma U87-MG tumours (Fig. 7B). The latter results, together with those obtained on *in vitro* TSPO binding, *in vivo* tissue distribution and PET imaging studies with specific and selective binding for TSPO, support the conclusion that [^18^F]CB251 shows favourable properties as a new TSPO-selective PET radiotracer compared with [^11^C]PBR28. To our knowledge, ours is the first example of an ^18^F-labelled TSPO PET imaging agent, namely, [^18^F]CB251, which is characterized by a 6,8-di-substituted imidazo[1,2-a]pyridine acetamide structure. [^18^F]CB251 was prepared with a satisfactory radiochemical yield and in tissue biodistribution experiments in mice, [^18^F]CB251 showed high accumulation in TSPO-enriched tissues such as the lung, heart, kidney, spleen and adrenal glands, whereas it exhibited low uptake in the liver and stomach. In the PET study, [^18^F]CB251 produced a clearly visible image of the inflammatory lesion with the highest target-to-background ratio at early imaging times in the brains of LPS-induced neuroinflammation model rats and displayed reasonable tumour uptake in the U87-MG xenograft model. These results suggest that [^18^F]CB251 is a promising TSPO PET imaging agent for neuroinflammation and TSPO-rich cancers in rodent models. It will be of interest to investigate the translation of these findings to other *in vivo* models and humans. The imidazopyridine alpidem binds both human and rodent TSPO with similar affinity^28^, although little data exists regarding the binding modes of this alpidem analogue to TSPO harbouring the natural Ala147Thr polymorphism. The recently published TSPO structure may help support further such structural investigations^29^,^30^,^31^.

## Methods

### Chemistry

Anhydrous solvents (*N*,*N*-dimethylformamide (DMF), tetrahydrofuran (THF), dichloromethane (CH_2_Cl_2_), acetonitrile (CH_3_CN), dimethylsulfoxide (DMSO), (diethylamino)sulfur trifluoride (DAST), (1,2-di(tosyloxy)ethane), sodium hydride (NaH) 60% dispersion in mineral oil, caesium carbonate (CsCO_3_), potassium carbonate (K_2_CO_3_), potassium iodide (KI) and *n*-tetrabutylammonium iodide (TBAI, *n*Bu_4_NI) were purchased from Sigma-Aldrich (Milan, Italy) and were used without further purification unless otherwise specified. TSPO ligand 2-(6,8-dichloro-2-(4-hydroxyphenyl)imidazo[1,2-a]pyridin-3-yl)-*N*,*N*-dipropylacetamide (**2**) was prepared according to the procedures described in the literature^11^. Melting points (mp) were obtained in open capillary tubes on a Büchi apparatus and are uncorrected. Elemental analyses were carried out with a Carlo Erba model 1106 elemental analyser. IR spectra were recorded on a PerkinElmer Fourier transform IR spectrophotometer and samples were prepared using KBr pellets. ^1^H- and ^19^F-NMR (300 MHz) spectra were recorded at room temperature on a Varian Mercury 300 MHz spectrometer. ^1^H and ^19^F chemical shifts are reported in δ values (ppm) downfield from tetramethylsilane and CFCl_3_ as internal standards, respectively. Electrospray ionisation mass spectrometry (ESI-MS) was performed on an Agilent 1100 LC-MSD trap system instrument. Silica gel 60 (70-230 mesh, Merck) was used for column chromatography. All reactions were carried out under a nitrogen atmosphere and the evolution was monitored using thin-layer chromatography (TLC silica gel 60 F254, Merck). H_2_^18^O was purchased from Taiyo Nippon Sanso Corporation (Japan). ^18^F-Fluoride was produced at Seoul National University Bundang Hospital by ^18^O(p,n)^18^F reaction through proton irradiation using a KOTRON-13 cyclotron (Samyoung Unitech Co., Ltd.). C18 plus Sep-Pak^®^ cartridges were purchased from Waters Corp. (U.S.A.). Purification was performed by HPLC using a Gilson 322 column (Waters, semi-Preparative Xterra RP-18, 10 μm, 10 × 250 mm) equipped with a UV detector (wavelength set at 254 nm) and a gamma-ray detector (Bioscan). A mixture of acetonitrile and water (60:40) was used as the mobile phase at a flow rate of 3 mL/min. The purified radiotracer and authentic compound were analysed in acetonitrile and water (60:40) as the mobile phase at a flow rate of 1 mL/min using Agilent 1260 infinity (U.S.A.). The HPLC system (Waters, Xterra RP-18, 4.6 × 250 mm, 5 μm) was equipped with an NaI radiodetector (Raytest) and a UV-detector. HPLC-grade solvents (J. T. Baker, U.S.A.) were used for HPLC purification after membrane filtration (Whatman, 0.22 μm). Radio-TLC was analysed on a Bioscan radio-TLC scanner (U.S.A.). All radioactivities were measured using a VDC-505 activity calibrator from Veenstra Instruments (Netherlands). To compare TSPO specificity between [^18^F]CB251 and [^11^C]PBR28, the authentic compound, PBR28 and desmethyl-PBR28 for labelling of [^11^C]PBR28 were purchased from Huayi Isotopes Co. (China). [^11^C]PBR28 was produced from desmethyl PBR28 and [^11^C]CH_3_OTf in a TRACERlab FX C Pro module (GE Healthcare) according to the procedures described in the literature^32^,^33^.

#### 2-(4-(6,8-Dichloro-3-(2-(dipropylamino)-2-oxoethyl)imidazo[1,2-a]pyridin-2-yl)-phenoxy)ethyl acetate (**3**)

Ethyl 2-bromoacetate (90 μL, 0.82 mmol), KI (0.18 g, 1.08 mmol), K_2_CO_3_ (0.4 g, 2.88 mmol) and *n*Bu_4_NI (0.05 g, 0.14 mmol) were added in order to a stirred solution of 2-(6,8-dichloro-2-(4-hydroxyphenyl)imidazo[1,2-a]pyridin-3-yl)-*N*,*N*-dipropylacetamide (**2**) (0.3 g, 0.72 mmol) in anhydrous DMF. The mixture was stirred overnight at room temperature and the solvent was then evaporated under reduced pressure. The residue was treated with water (50 mL) and the mixture was extracted with ethyl acetate (3 × 20 mL). The organic layer was dried over Na_2_SO_4_ and completely dried under vacuum. The crude product was purified by silica gel column chromatography [light petroleum ether:ethyl acetate = 1:1 (v/v)] to yield **3** (0.28 g, 78%) as a white solid: mp 105–108 °C; IR (KBr): 1736, 1645 cm^−1^; ^1^H NMR (CDCl_3_) δ: 0.74 (t, *J* = 7.4 Hz, 3H, CH_3_), 0.87 (t, *J* = 7.4 Hz, 3H, CH_3_), 1.4–1.6 (m, 4H, CH_2_), 2.13 (s, 3H, CH_3_COO), 3.10 (t, *J* = 7.7 Hz, 2H, CH_2_NCO), 3.30 (t, *J* = 7.7 Hz, 2H, CH_2_NCO), 4.07 (s, 2H, CH_2_CO), 4.23 (t, 2H, *J* = 5.0 Hz, CH_2_O), 4.46 (t, 2H, *J* = 5.0 Hz, CH_2_OCO), 7.02 (d, *J* = 9.0 Hz, 2H, Ar), 7.27 (d, *J* = 2.0 Hz, 1H, Ar), 7.61 (d, *J* = 9.0 Hz, 2H, Ar), 8.29 (d, *J* = 2.0 Hz, 1H, Ar); MS (ESI) *m*/*z* 529.2 (M + Na) ^+^ . Anal. (C_25_H_29_Cl_2_N_3_O_4_) C, H, N.

#### 2-(2-(4-(2-Hydroxyethoxy)phenyl)-6,8-dichloroimidazo[1,2-a]pyridin-3-yl)-N,N-dipropylacetamide (**4**)

A solution of CsCO_3_ (0.18 g, 0.56 mmol) in H_2_O (2 mL) was added to a stirred solution of 2-(4-(6,8-dichloro-3-(2-(dipropylamino)-2-oxoethyl)imidazo[1,2-a]pyridin-2-yl)-phenoxy)ethyl acetate (**3**) (0.2 g, 0.4 mmol) in MeOH (4 mL). The reaction mixture was stirred overnight at room temperature and the solvent was then removed under reduced pressure. The residue was treated with water (50 mL) and the mixture was extracted with ethyl acetate (3 × 20 mL). The organic layer was dried over Na_2_SO_4_ and completely dried under vacuum. The crude product was purified by silica gel column chromatography [light petroleum ether:ethyl acetate = 1:1 (v/v)] to yield **4** (0.15 g, 80%) as an orange solid: mp 175–177 °C; IR (KBr): 3434, 1636 cm^−1^; ^1^H NMR (CDCl_3_) δ: 0.72 (t, *J* = 7.4 Hz, 3H, CH_3_), 0.85 (t, *J* = 7.4 Hz, 3H, CH_3_), 1.4–1.6 (m, 4H, CH_2_), 3.11 (t, *J* = 7.4 Hz, 2H, CH_2_NCO), 3.28 (t, *J* = 7.4 Hz, 2H, CH_2_NCO), 3.99 (t, *J* = 7.4 Hz, 2H, CH_2_OH), 4.05 (s, 2H, CH_2_CO), 4.13 (t, *J* = 7.4 Hz, 2H, CH_2_OAr), 7.00 (d, *J* = 8.8 Hz, 2H, Ar), 7.28 (d, *J* = 1.6 Hz, 1H, Ar), 7.58 (d, *J* = 8.8 Hz, 2H, Ar), 8.28 (d, *J* = 1.6 Hz, 1H, Ar); MS (ESI) *m*/*z* 487.0 (M + Na) ^+^ . Anal. (C_23_H_27_Cl_2_N_3_O_3_) C, H, N.

#### 2-(4-(6,8-Dichloro-3-(2-(dipropylamino)-2-oxoethyl)imidazo[1,2-a]pyridin-2-yl)phenoxy)ethyl 4-methylbenzenesulfonate (**5**)

A suspension of NaH (60% in mineral oil, 34 mg, 1.44 mmol) was added dropwise to a solution of 2-(6,8-dichloro-2-(4-hydroxyphenyl)imidazo[1,2-a]pyridin-3-yl)-*N*,*N*-dipropylacetamide (**2**) (0.2 g, 0.48 mmol) in anhydrous THF (5 mL). The reaction mixture was stirred at 0 °C for 10 min and then allowed to warm up to room temperature. Next, a solution of 1,2-di(tosyloxy)ethane (0.53 g, 1.44 mmol) in anhydrous THF (2 mL) was added dropwise. After an additional 10 min, the reaction mixture was refluxed and stirred overnight. The solvent was removed by vacuum and the residue was treated with 1 N HCl (25 mL). Afterward, the mixture was extracted with CH_2_Cl_2_ (3 × 20 mL) and the organic layer was dried over Na_2_SO_4_ and completely dried under vacuum. The crude product was purified by silica gel column chromatography [CH_2_Cl_2_:MeOH = 95:5 (v:v)] to yield **5** (0.23 g, 78%) as a light yellow solid: mp 123–125 °C; IR (KBr): 1646, 1613 cm^−1^; ^1^H NMR (CDCl_3_) δ: 0.73 (t, *J* = 7.4 Hz, 3H, CH_3_), 0.75 (t, *J* = 7.4 Hz, 3H, CH_3_), 1.4–1.6 (m, 4H, CH_2_), 2.45 (s, 3H, CH_3_Ar), 3.09 (t, *J* = 7.7 Hz, 2H, CH_2_NCO), 3.28 (t, *J* = 7.7 Hz, 2H, CH_2_NCO), 4.04 (s, 2H, CH_2_CO), 4.67 (dd, *J* = 8.8, 8.8 Hz, 2H, CH_2_O), 4.88 (dd, *J* = 8.8, 8.8 Hz, 2H, CH_2_OTs), 6.90 (d, *J* = 8.8 Hz, 1H, Ar), 7.3–7.4 (m, 3H, Ar), 7.55 (d, *J* = 8.8 Hz, 1H, Ar), 7.7–7.9 (m, 4H, Ar), 8.27 (d, *J* = 1.8 Hz, 1H, Ar); MS (ESI) *m*/*z* 640.2 (M + Na) ^+^ . Anal. (C_30_H_33_Cl_2_N_3_O_5_S) C, H, N.

#### 2-(2-(4-(2-Fluoroethoxy)phenyl)-6,8-dichloroimidazo[1,2-a]pyridin-3-yl)-N,N-dipropylacetamide (**1**) (CB251)

A solution of compound **4** (0.1 g, 0.22 mmol) in anhydrous CH_2_Cl_2_ (10 mL) was added to a solution of DAST (0.037 g, 0.23 mmol) in anhydrous CH_2_Cl_2_ (10 mL). The reaction mixture was stirred at room temperature for 3 h. After the solvent was evaporated under reduced pressure, the residue was treated with water (50 mL) and the mixture was extracted with ethyl acetate (3 × 20 mL). The organic layer was dried over Na_2_SO_4_ and completely dried under vacuum. The residue was purified by silica gel column chromatography [light petroleum ether:ethyl acetate = 7:3 (v/v) as eluent] to obtain **1** (51 mg, 50%) as a white solid: mp 158–159 °C; IR (KBr): 1637 cm^−1^; ^1^H NMR (CDCl_3_) δ: 0.72 (t, *J* = 7.4 Hz, 3H, CH_3_), 0.85 (t, *J* = 7.4 Hz, 3H, CH_3_), 1.4–1.6 (m, 4H, CH_2_), 3.08 (t, *J* = 7.7 Hz, 2H, CH_2_NCO), 3.28 (t, *J* = 7.7 Hz, 2H, CH_2_NCO), 4.05 (s, 2H, CH_2_CO), 4.26 (dt, *J*^3^_H-F_ = 27.8 Hz and *J*^3^_H-H_ = 4.1 Hz, 2H, OCH_2_CH_2_F), 4.78 (dt, *J*^2^_H-F_ = 47.3 Hz and *J*^3^_H-H_ = 4.1 Hz, 2H, CH_2_F), 7.01 (d, *J* = 8.8 Hz, 2H, Ar), 7.27 (d, *J* = 1.6 Hz, 1H, Ar), 7.59 (d, *J* = 8.8 Hz, 2H, Ar), 8.28 (d, *J* = 1.6 Hz, 1H, Ar); ^19^F-NMR (CDCl_3_) δ: 130.03; MS (ESI) *m*/*z* 489.2 (M + Na)^+^. Anal. (C_23_H_26_Cl_2_FN_3_O_2_) C, H, N.

### Radiochemical synthesis of [^18^F]1 ([^18^F]CB251)

The radiotracer, [^18^F]CB251, was synthesized in a single-step radiolabelling procedure by nucleophilic aliphatic substitution using tosylate precursor **5** with fluorine-18. Briefly, ^18^F was obtained by the ^18^O(p,n)^18^F nuclear reaction from [^18^O]water. Fluorine-18 was isolated from the enriched water by trapping in a Chromafix-HCO_3_ cartridge (pre-activated with 2 mL of ethanol and 5 mL of water) and then eluted with methanol:water (1:0.1 mL) dissolved TBAHCO_3_ or K_2.2.2._/K_2_CO_3_. The eluted solution with fluorine-18 was dried by azeotropic distillation with acetonitrile (0.3 mL) under a nitrogen stream (× 2) and the tosylate precursor **5** (3.0 mg) in various reaction solvents (0.4 mL), such as CH_3_CN, DMF, DMSO or *tert*-butanol, was subsequently added. The reaction mixture was heated to the desired temperature for 10 or 30 min. The reaction mixture was cooled to room temperature and was then diluted with 10 mL of water. This solution was loaded into a C18 plus Sep-Pak cartridge, washed with 10 mL of water and eluted with 1.5 mL of CH_3_CN. After dilution with 1.5 mL of water, the combined solution was separated by a semi-preparative HPLC system using a UV detector at 254 nm and a gamma-ray detector. The product fraction was collected after approximately 20 min (Fig. S1). The fraction of [^18^F]**1** collected from the HPLC system was diluted with 20 mL of water. The solution was exchanged to 10% EtOH/saline solution by a C18 plus Sep-Pak cartridge to remove the HPLC solvent.

### Log D determination

The log D value was measured four times by mixing a solution of [^18^F]CB251 (approximately 0.74 MBq) or [^11^C]PBR28 (approximately 1.5 MBq) in 5% ethanol:saline (10 μL) with sodium phosphate buffer (0.15 M, pH 7.4, 5.0 mL) and *n*-octanol (5.0 mL) in a test tube. After vortexing for 1 min, each tube was then stored for 3 min at room temperature and the phases were separated. Samples of each phase (100 μL) were counted for radioactivity. Log D is expressed as the logarithm of the ratio of the counts from *n*-octanol versus that of the sodium phosphate buffer.

### Stability of [^18^F]CB251 in human serum

The *in vitro* stability of [^18^F]CB251 was assayed by monitoring both the radio-TLC and the radio-HPLC profiles to determine its radiochemical purity. To assess the *in vitro* serum stability, 100 μL of [^18^F]CB251 in 10% EtOH/saline was incubated with 0.5 mL of human serum from a healthy volunteer for 2 h at 37 °C and the solution was analysed at 0, 10, 30, 60 and 120 min by a radio-TLC scanner using MeOH-CH_2_Cl_2_ (5:95) as the developing solvent. Separately, 100 μL of [^18^F]CB251 in 10% EtOH/saline was also incubated with 0.9 mL of human serum from a healthy volunteer for 2 h at 37 °C. Aliquots (200 μL) were taken from the sample at time points of 0, 30, 60 and 120 min and mixed with absolute acetonitrile (200 μL). The mixture was centrifuged (3500 rpm, 5 min) to precipitate out serum proteins. The resulting supernatant including the authentic CB251 (1.0 mg/2.0 mL of CH_3_CN, 20 μL) was filtered through a 0.45 μm GH Polypro (GHP) membrane Disc filter before HPLC analysis to determine the fraction of intact radiotracer. Samples were analysed by HPLC (Waters, Xterra RP-18, 5 μm, 4.6 × 250 mm; eluant: 60% CH_3_CN/H_2_O; flow rate: 1 mL/min; guard column 10 × 10 mm).

### *In vitro* TSPO binding assays

Binding affinity and selectivity to the 18-kDa translocator protein TSPO and to CBR were assessed using *in vitro* receptor binding assays. These experiments were carried out as described by Denora *et al*^34^.

### *Ex vivo* tissue distribution in normal mice

Tissue distribution studies were performed in normal male ICR mice (7 weeks, 25-30 g) with tail-vein administration of [^18^F]CB251 (0.74 MBq/mouse) dissolved in 10% ethanol in saline to a final volume of 200 μL. The animals (*n* = 5 for each time point) were euthanised with diethyl ether at 5, 15, 30, 60 and 120 min post injection. After the mice were euthanised, blood and other organs such as the heart, liver, lung, stomach, spleen, kidney, intestine, muscle, brain, adrenal and femur were removed, weighed and counted for radioactivity in a NaI (Tl) well counter (Wizard^TM^3”, PerkinElmer). The injected dose was calculated from standards prepared from the injection solution. The accumulated radioactivity of all tissues is presented as the percentage of the injected dose per gram of tissue (%ID/g). All animal studies were performed with the approval of the Institutional Animal Care and Use Committee of the Seoul National University Bundang Hospital (SNUBH) and the methods were carried out in accordance with the approved guidelines.

### LPS-induced neuroinflammation rat model

Male Sprague-Dawley rats weighing 200–250 g were used for the brain inflammation model and neuroinflammation was induced using lipopolysaccharide (LPS; Sigma-Aldrich, St. Louis, MO, U.S.A.) as previously described^22^. In all rats used for sequential PET scans, microglia activation between the ipsilateral and contralateral areas was determined by CD68 immunohistochemical staining.

### Human glioblastoma U87-MG xenograft model

U87-MG cells (ATCC) were cultured in Dulbecco’s modified Eagle’s medium supplemented with 4 mM L-glutamine, 4,500 mg/L glucose, 1 mM sodium pyruvate, 1.5 g/mL sodium bicarbonate, 10% FBS and antibiotic-antimycotic (Gibco, Grand Island, NY, USA) at 37 °C in a humidified atmosphere of 5% CO_2_. U87-MG cells were cultured for 2–3 days without changing the medium and were harvested with trypsin-EDTA in PBS. Male Balb/c nu/nu mice, obtained at 6–7 weeks of age (Orient Bio Inc., Seongnam, Korea), were subcutaneously injected in the right shoulder with 5 × 10^6^ U87-MG cells suspended in 100 μL of 100 mM PBS (pH 7.4). Animal protocols were approved by the Seoul National University Bundang Hospital Animal Care and Use Committee and the methods were carried out in accordance with the approved guidelines. We also performed calliper measurements of the longest perpendicular tumour diameters.

### Small animal PET imaging study

**Figure 8:**
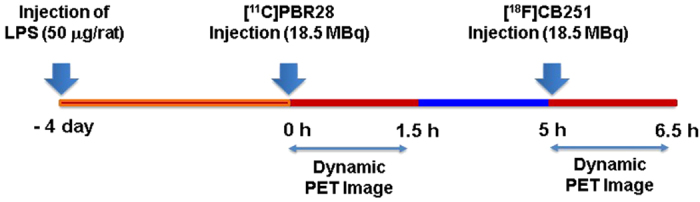
Experimental design of the PET imaging study.

Five LPS-induced neuroinflammation rats (219.2 ± 14.1 g) were used for brain PET imaging at 4 days post-LPS injection. Before the scan, the rats were anesthetized with 2% isoflurane in a 7:3 mixture of N_2_/O_2_. PET imaging of the rat brain was performed in a dedicated small animal PET/CT (NanoPET/CT, Bioscan, Inc., U.S.A.) with a 10-cm axial field-of-view (FOV) and a 12-cm transaxial FOV. PET acquisition in the list-mode was concomitantly started with the intravenous injection of either [^11^C]PBR28 or [^18^F]CB251 and was performed for 90 min, followed by a CT scan of the head or body for attenuation correction. The first exam was performed with [^11^C]PBR28 (approximately 18.5 MBq) and the second exam followed 3.5 h later with [^18^F]CB251 (approximately 18.5 MBq) in same rat models (Fig. 8). To determine the TSPO selectivity and TSPO specificity of the PET images, flumazenil (5 mg/kg intravenously) was administered at 15 min post [^18^F]CB251 injection via jugular catheter. Subsequently, PBR28 (5 mg/kg intravenously) was injected 30 min later via jugular catheter in the same LPS-induced neuroinflammation rat model. Three human glioblastoma U87-MG-bearing mice were used for TSPO-rich cancer PET imaging. PET scans of the mice, anesthetized with 2% isoflurane, were performed with a 9.3-cm axial, 8.4-cm trans-axial FOV scanner. At the end of each study, the list-mode data were sorted into a dynamic scan consisting of 54 frames for 90 min. The acquired images were reconstructed by the 3-D Adjoints Monte Carlo method, which included scatter and random corrections. A region of interest (ROI) with a 2-mm radius was delineated in the ipsilateral lesioned striatum by the intensely visualized region in the summed image of all the frames. Regional uptake of radioactivity was decay-corrected to the injection time and was expressed as the standardized uptake value (SUV), which was normalized to the injected radioactivity and animal body weight. Analysis of the PET images was performed using PMOD software v.3.1 (PMOD Technologies Ltd., Zurich, Switzerland). The time activity curve (TAC) representing the variation in radioligand concentration according to the time-course was estimated for the ipsilateral striatum. The binding potential of the specifically bound radioligand relative to the non-displaceable radioligand in tissue (BP_ND_) for both [^11^C]PBR28 and [^18^F]CB251 on the ipsilateral striatum (right) was calculated with Logan plot with reference tissue input model using the contralateral striatum (left) as the reference region^35^,^36^.

### Immunohistochemical staining

Immunohistochemical staining was performed using serial sections of brain and tumours that had undergone PET/CT imaging. Briefly, the isolated brains and tumours were embedded in a tissue-freezing medium (Triangle Biomedical Sciences, Durham, NC, USA), frozen and consecutively cryosectioned in 10-μm segments using a Cryocut Microtome (CM3050S, Leica, Solms, Germany). The tissue sections were thaw-mounted onto silane-coated microscope slides (Muto Pure Chemicals Co., Tokyo, Japan), dried in an aeration room and stored at −80 °C until use. The tissue sections were rinsed with phosphate-buffered saline (PBS) and fixed with 4% paraformaldehyde for 20 min at room temperature. After an additional series of washes with PBS, the sections were cleared with 3% sodium deoxycholate solution for 2 h at room temperature, blocked with 20% normal goat serum in 1% BSA-PBS for 2 h at 37 °C and incubated with the primary antibodies at 4 °C overnight (>17 h). A total of 100 μL DAPI solution (1 μg/mL) was added to completely cover the section and incubated for 5 min at room temperature. Specific antibodies against macrophage marker CD68 (1:150, Abd Serotec, MCA1957) were used on the brain and tumour sections. Subsequently, the slides were rinsed three times with PBS and incubated with secondary antibodies (1:400, Alexa Fluor 488 labelled, Life Technologies, Carlsbad, CA, USA) for 2 h at 4 °C. Then, the slides were washed several times with PBS, counterstained with Hoechst 33342 dye (1:750, Life Technologies) and mounted with Prolong Gold antifade reagent (Life Technologies). Images were captured with the use of a confocal microscope (A1, Leica, Solms, Germany).

### Statistical analysis

The data are expressed as the mean ± standard error of the mean (SEM) and were analysed using the Mann–Whitney *U*-test. Statistical significance was defined as *p* < 0.05. Statistical analyses were performed using SPSS 12 software (SPSS, Inc., Chicago, IL, USA).

## Additional Information

**How to cite this article**: Perrone, M. *et al.* A Novel PET Imaging Probe for the Detection and Monitoring of Translocator Protein 18 kDa Expression in Pathological Disorders. *Sci. Rep.* **6**, 20422; doi: 10.1038/srep20422 (2016).

## Supplementary Material

Supplementary Information

## Figures and Tables

**Figure 1:**
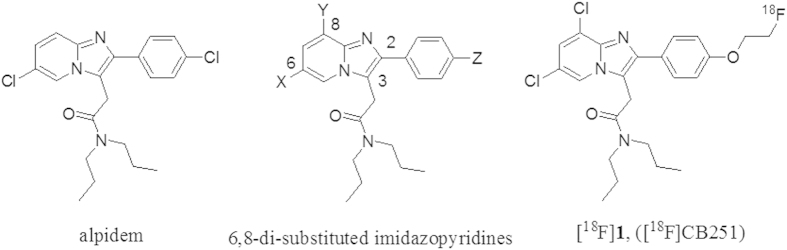
Structures of imidazopyridine TSPO ligands.

**Table 1:** Radiosynthesis of [^18^F]CB251 under various conditions^a^.

entry	base	solvent	time (min)	temp. (°C)	RCY (%)^b^
1	*n*Bu_4_NHCO_3_ (0.8 equiv.)	CH_3_CN	10	120	<1
2	*n*Bu_4_NHCO_3_ (0.8 equiv.)	DMSO	10	140	<1
3	*n*Bu_4_NHCO_3_ (0.8 equiv.)	DMF	10	140	<1
4	*n*Bu_4_NHCO_3_ (0.8 equiv.)	*tert*-BuOH	10	120	4.1 ± 1.5^b^
5	*n*Bu_4_NHCO_3_ (0.8 equiv.)	*tert*-BuOH	30	120	4.0 ± 1.2^b^
6	*n*Bu_4_NHCO_3_ (2.0 equiv.)	*tert*-BuOH	10	120	17.3 ± 4.9^b^
7	*n*Bu_4_NHCO_3_ (4.0 equiv.)	*tert*-BuOH	10	120	28.1 ± 7.2^c^
8	*n*Bu_4_NHCO_3_ (8.0 equiv.)	*tert*-BuOH	10	120	10.6 ± 4.8^b^
9	K_2.2.2_/K_2_CO_3_ (5 mg/0.8 equiv.)	*tert*-BuOH	10	120	14.7 ± 4.2^b^
10	K_2.2.2_/K_2_CO_3_ (5 mg/2.0 equiv.)	*tert*-BuOH	10	120	1.7 ± 0.5^b^

^a^The reaction was carried out using different equivalents of base relative to the precursor (3.0 mg) in 0.4 mL of solvent.

^b^The radiochemical yield (RCY) was determined by radio-TLC (*n* = 3).

^c^*n* = 21.

**Table 2:** *In vitro* binding affinity.

Compound	K_i_(nM) for TSPO^a^	K_i_(nM) for CBR^a^
CB251	0.27 ± 0.09	>10^5^
PK 11195	1.38 ± 0.42; 1.4 ± 0.4^c^	>10^5^
PBR28	6.1 ± 6.4^c^	–
flunitrazepam^b^	–	5.11 ± 0.87

^a^Affinity of compounds determined by displacement of [^3^H]PK 11195 and [^3^H]flunitrazepam from rat cerebrocortical samples. The data are expressed as the mean values of three separate experiments performed in duplicate, which differed by less than 10%.

^b^Flunitrazepam, a selective ligand for CBR, was used for comparison.

^c^According to Ref. 25.

**Table 3:** *Ex vivo* tissue biodistribution of [^18^F]CB251 in normal ICR mice^a^.

Organ	Time point				
	5 min	15 min	30 min	60 min	120 min
Blood	4.39 ± 0.78	2.17 ± 0.40	1.24 ± 0.26	0.60 ± 0.12	0.64 ± 0.26
Heart	29.49 ± 2.36	24.16 ± 4.67	23.11 ± 5.16	10.47 ± 4.50	8.62 ± 4.57
Liver	4.69 ± 0.66	6.67 ± 1.10	8.19 ± 1.59	6.80 ± 2.64	7.17 ± 2.99
Lung	106.4 ± 8.61	65.70 ± 11.3	38.36 ± 3.97	17.69 ± 5.77	13.22 ± 6.10
Stomach	1.86 ± 0.48	2.13 ± 0.77	1.84 ± 0.49	0.98 ± 0.41	1.97 ± 0.95
Spleen	17.63 ± 3.38	25.11 ± 2.16	23.27 ± 3.52	14.24 ± 4.47	13.82 ± 5.17
Kidney	22.17 ± 1.63	24.55 ± 5.11	23.19 ± 5.68	14.55 ± 5.74	15.60 ± 8.55
L. Intestine	1.54 ± 0.19	1.53 ± 0.13	1.69 ± 0.21	1.37 ± 0.56	2.09 ± 1.07
S. Intestine	3.30 ± 0.73	4.54 ± 0.60	4.43 ± 0.90	3.43 ± 1.35	3.78 ± 1.96
Muscle	0.83 ± 0.34	1.19 ± 0.22	2.01 ± 0.34	1.52 ± 0.59	1.45 ± 0.65
Brain	2.89 ± 0.23	1.94 ± 0.24	1.39 ± 0.13	0.63 ± 0.24	0.53 ± 0.23
Adrenal	19.06 ± 5.48	22.60 ± 5.00	19.73 ± 3.42	12.69 ± 5.62	14.78 ± 8.26
Femur	1.19 ± 0.50	1.25 ± 0.41	1.94 ± 0.36	1.41 ± 0.64	1.49 ± 0.80

^a^The data are expressed as mean values and standard deviations (*n* = 5).

## References

[b1] Venneti S., Lopresti B. J. & Wiley C. A. The peripheral benzodiazepine receptor (Translocator protein 18 kDa) in microglia: From pathology to imaging. Prog. Neurobiol. 80, 308–22 (2006). 17156911 10.1016/j.pneurobio.2006.10.002 PMC1849976

[b2] McGeer P. L., Itagaki S., Boyes B. E. & McGeer E. G. Reactive microglia are positive for HLA-DR in the substantia nigra of Parkinson’s and Alzheimer’s disease brains. Neurology 38, 1285–91 (1988). 3399080 10.1212/wnl.38.8.1285

[b3] Bauer J., Sminia T., Wouterlood F. G. & Dijkstra C. D. Phagocytic activity of macrophages and microglial cells during the course of acute and chronic relapsing experimental autoimmune encephalomyelitis. J. Neurosci. Res. 38, 365–75 (1994). 7932870 10.1002/jnr.490380402

[b4] Schweitzer P. J., Fallon B. A., Mann J. J. & Kumar J. S. PET tracers for the peripheral benzodiazepine receptor and uses thereof. Drug Discov. Today 15, 933–42 (2010). 20800696 10.1016/j.drudis.2010.08.012

[b5] Maaser K., Grabowski P., Sutter A. P., Höpfner M. & Foss H. Overexpression of the peripheral benzodiazepine receptor is a relevant prognostic factor in stage III colorectal cancer. Clin. Cancer. Res. 8, 3205–9 (2002). 12374690

[b6] Veenman L. *et al.* Peripheral-type benzodiazepine receptor density and *in vitro* tumorigenicity of glioma cell lines. Biochem. Pharmacol. 68, 689–98 (2004). 15276076 10.1016/j.bcp.2004.05.011

[b7] Le Fur G. *et al.* Peripheral benzodiazepine binding sites: effect of PK11195, 1-(2-chlorophenyl)-*N*-(1-methylpropyl)-3-isoquinolinecarboxamide I. *In vitro* studies. Life Sci. 32, 1839–47 (1983). 6300588 10.1016/0024-3205(83)90062-0

[b8] Marangos P. L., Pate J., Boulenger J. P. & Clark-Rosenberg R. Characterization of peripheral-type benzodiazepine binding sites in brain using [^3^H]Ro 5-4864. Mol. Pharmacol. 22, 26–32 (1982). 6289073

[b9] Romeo E. *et al.* 2-Aryl-3-indoleacetamides (FGIN-1): a new class of potent and specific ligands for the mitochondrial DBI receptor (MDR). J. Pharmacol. Exp. Ther. 262, 971–78 (1992). 1326631

[b10] Imaizumi M. *et al.* Brain and whole-body imaging in nonhuman primates of [^11^C]PBR28, a promising PET radioligand for peripheral benzodiazepine receptors. NeuroImage 39, 1289–98 (2008). 18024084 10.1016/j.neuroimage.2007.09.063 PMC2275117

[b11] Denora N. *et al.* 2-Phenyl-imidazo[1,2-a]pyridine compounds containing hydrophilic groups as potent and selective ligands for peripheral benzodiazepine receptors: synthesis, binding affinity and electrophysiological studies. J. Med. Chem. 51, 6876–88 (2008). 18834105 10.1021/jm8006728

[b12] Laquintana V. *et al.* *N*-Benzyl-2-(6,8-dichloro-2-(4-chlorophenyl)imidazo[1,2-a]pyridin-3-yl)-N-(6-(7-nitrobenzo[c][1,2,5]oxadiazol-4-ylamino)hexyl)acetamide as a new fluorescent probe for peripheral benzodiazepine receptor and microglial cell visualization. Bioconjugate Chem. 18, 1397–1407 (2007). 10.1021/bc060393c 17722875

[b13] Denora N. *et al.* New fluorescent probes targeting the mitochondrial-located translocator protein 18 kDa (TSPO) as activated microglia imaging agents. Pharm. Res. 28, 2820–32 (2011). 21818711 10.1007/s11095-011-0552-0

[b14] Laquintana V. *et al.* Peripheral benzodiazepine receptor ligand–PLGA polymer conjugates potentially useful as delivery systems of apoptotic agents. J. Control Release. 137, 185–95 (2009). 19374931 10.1016/j.jconrel.2009.04.007

[b15] Denora N. *et al.* *In vitro* targeting and imaging the translocator protein TSPO 18-kDa through G(4)-PAMAM-FITC labeled dendrimer. J. Control Release. 172, 1111–25 (2013). 24096015 10.1016/j.jconrel.2013.09.024

[b16] Pappata S. *et al.* PET study of carbon-11-PK11195 binding to peripheral type benzodiazepine sites in glioblastoma: a case report. J. Nucl. Med. 32, 1608–10 (1991). 1651383

[b17] Chauveau F., Boutin H., Van Camp N., Dollé F. & Tavitian B. Nuclear imaging of neuroinflammation: a comprehensive review of [^11^C]PK11195 challengers. Eur. J. Nucl. Med. Mol. Imaging 35, 2304–19 (2008). 18828015 10.1007/s00259-008-0908-9

[b18] Sekimata K. *et al.* Radiosynthesis and *in vivo* evaluation of *N*-[^11^C]methylated imidazopyridineacetamides as PET tracers for peripheral benzodiazepine receptors. Nucl. Med. Biol. 35, 327–34 (2008). 18355688 10.1016/j.nucmedbio.2007.12.005

[b19] Hatano K. *et al.* Radiosynthesis and *in vivo* evaluation of two imidazopyridineacetamides, [^11^C]CB184 and [^11^C]CB190, as a PET tracer for 18 kDa translocator protein: direct comparison with [^11^C](R)-PK11195. Ann. Nucl. Med. 29, 325–35 (2015). 25616581 10.1007/s12149-015-0948-8 PMC4835529

[b20] Price G. W. *et al.* *In vivo* binding to peripheral benzodiazepine binding sites in lesioned rat brain: comparison between [^3^H]PK11195 and [^18^F]PK14105 as markers for neuronal damage. J. Neurochem. 55, 175–85 (1990). 2355218 10.1111/j.1471-4159.1990.tb08836.x

[b21] Yu W., Wang E., Voll R. J., Miller A. H. & Goodman M. M. Synthesis, fluorine-18 radiolabeling and *in vitro* characterization of 1-iodophenyl-*N*-methyl-*N*-fluoroalkyl-3-isoquinoline carboxamide derivatives as potential PET radioligands for imaging peripheral benzodiazepine receptors. Bioorg. Med. Chem. 16, 6145–55 (2008). 18472268 10.1016/j.bmc.2008.04.046

[b22] Moon B. S. *et al.* [^18^F]Fluoromethyl-PBR28 as a potential radiotracer for TSPO: preclinical comparison with [^11^C]PBR28 in a rat model of neuroinflammation. Bioconjugate Chem. 25, 442–50 (2014). 10.1021/bc400556h 24400917

[b23] Dedeurwaerdere S. *et al.* PET imaging of brain inflammation during early epileptogenesis in a rat model of temporal lobe epilepsy. EJNMMI Res. 2, 60 (2012). 23136853 10.1186/2191-219X-2-60 PMC3570346

[b24] Callaghan P. D. *et al.* Comparison of *in vivo* binding properties of the 18-kDa translocator protein (TSPO) ligands [^18^F]PBR102 and [^18^F]PBR111 in a model of excitotoxin-induced neuroinflammation. Eur. Nucl. Med. Mol. Imaging 42, 138–51 (2015). 10.1007/s00259-014-2895-3 25231248

[b25] Kreisl C. W. *et al.* Comparison of [^11^C]-(R)-PK 11195 and [^11^C]PBR28, two radioligands for translocator protein (18 kDa) in human and monkey: Implications for positron emission tomographic imaging of this inflammation biomarker. NeuroImage 49, 2924–32 (2010). 19948230 10.1016/j.neuroimage.2009.11.056 PMC2832854

[b26] Papadopoulos V. *et al.* Translocator protein (18 kDa): new nomenclature for the peripheral-type benzodiazepine receptor based on its structure and molecular function. Trends Pharmacol. Sci. 27, 402–9 (2006). 16822554 10.1016/j.tips.2006.06.005

[b27] Wu C. *et al.* Longitudinal PET imaging of muscular inflammation using ^18^F-DPA-714 and ^18^F-Alfatide II and differentiation with tumors. Theranostics 4, 546–55 (2014). 24672585 10.7150/thno.8159 PMC3966057

[b28] Langer S. Z. *et al.* Selectivity for omega-receptor subtypes as a strategy for the development of anxiolytic drugs. Pharmacopsychiatry. 23, 103–7 (1990). 1974068 10.1055/s-2007-1014544

[b29] Jaremko L. *et al.* Structure of the mitochondrial translocator protein in complex with a diagnostic ligand. Science. 343, 1363–6 (2014). 24653034 10.1126/science.1248725 PMC5650047

[b30] Guo Y. *et al.* Structure and activity of tryptophan-rich TSPO proteins. Science. 347, 551–5 33 (2015). 25635100 10.1126/science.aaa1534 PMC4341906

[b31] Li F. *et al.* Crystal structures of translocator protein (TSPO) and mutant mimic of a human polymorphism. Science. 347, 555–8 (2015). 25635101 10.1126/science.1260590 PMC5125025

[b32] Breisl C. W. *et al.* Comparison of [^11^C]-(R)-PK 11195 and [^11^C]PBR28, two radioligands for translocator protein (18 kDa) in human and monkey: Implications for positron emission tomographic imaging of this inflammation biomarker. NeuroImage 49, 2924–32 (2010). 19948230 10.1016/j.neuroimage.2009.11.056 PMC2832854

[b33] Moon B. S. *et al.* Development of an additive [^11^C]CO_2_ target system in the KOTRON-13 cyclotron and its application for [^11^C]radiopharmaceutical production. Nucl. Instrum. Meth. Phys. Res. B 356–357, 1–7 (2015).

[b34] Denora N. *et al.* Translocator protein (TSPO) ligand-Ara-C (cytarabine) conjugates as a strategy to deliver antineoplastic drugs and to enhance drug clinical potential. Mol. Pharm. 6, 2255–69 (2010). 20958082 10.1021/mp100235w

[b35] Innis R. B. *et al.* Consensus nomenclature for *in vivo* imaging of reversibly binding radioligands. J Cereb Blood Flow Metab. 27, 1533–9 (2007). 17519979 10.1038/sj.jcbfm.9600493

[b36] Logan J. *et al.* Graphical analysis of reversible radioligand binding from time-activity measurements applied to [N-^11^C-methyl]-(-)-cocaine PET studies in human subjects. J Cereb Blood Flow Metab. 10, 740–7 (1990). 2384545 10.1038/jcbfm.1990.127

