# Supplementary material for: A Novel PET Imaging Probe for the Detection and Monitoring of Translocator Protein 18 kDa Expression in Pathological Disorders
Source: Sci Rep. 2016 Feb 8;6:20422. doi: 10.1038/srep20422 (PMC4745082; doi:10.1038/srep20422)
Supplement: Supplementary Information [file srep20422-s1.doc]

**Supplementary Information**

A Novel PET Imaging Probe for the Detection and Monitoring of Translocator Protein 18 kDa Expression in Pathological Disorders

*Mara Perrone,^§^ Byung Seok Moon,^§^ Hyun Soo Park, Valentino Laquintana, Jae Ho Jung, Annalisa Cutrignelli, Angela Lopedota, Massimo Franco, Sang Eun Kim, Byung Chul Lee,* Nunzio Denora **

^§^Mara Perrone and Byung Seok Moon contributed equally to this study.

1. **HPLC chromatogram (Figure S1 and S2)**
2. ***In vitro* stability of [^18^F]CB251 in human serum (Figure S3)**

**I. HPLC chromatogram**


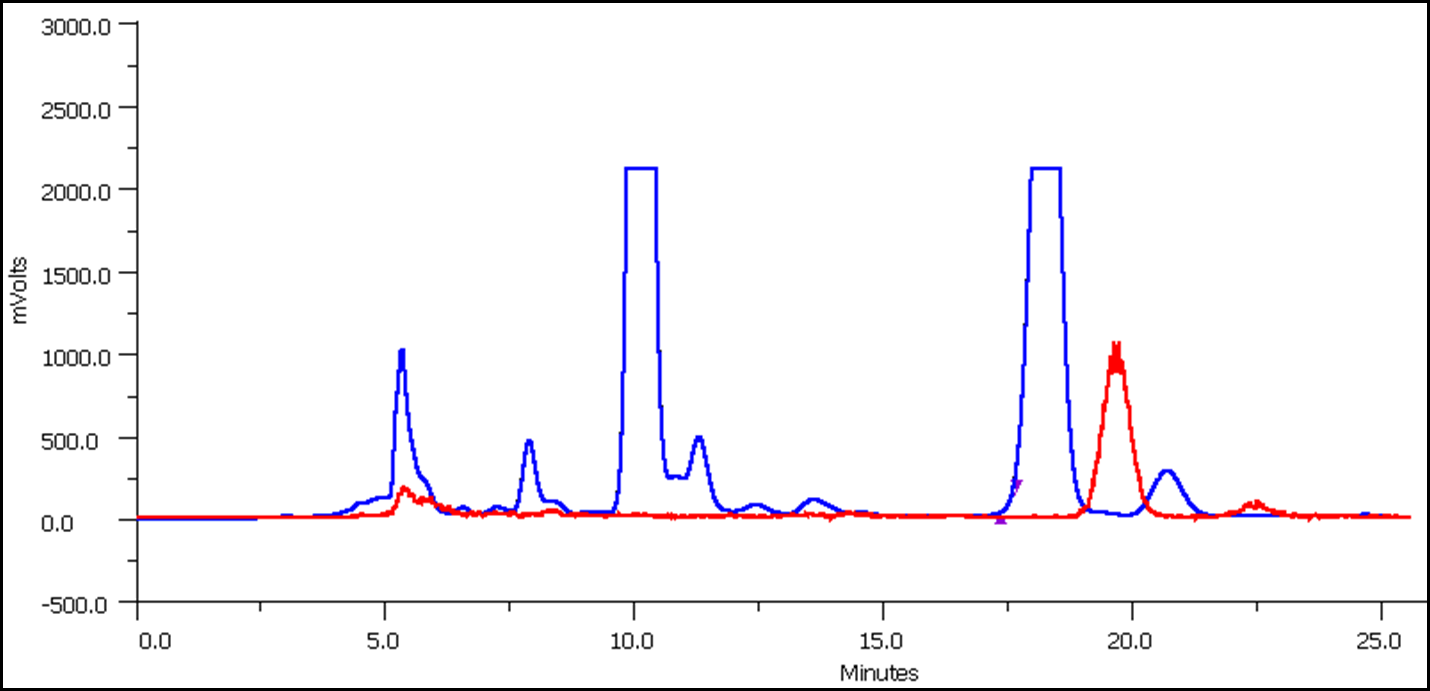


**Fig. S1** HPLC chromatogram of the reaction mixture; HPLC semi-preparative column (Waters, Xterra RP-18, 10 μm, 10 x 250 mm) with a guard cartridge (Phenomenex, 10 x 10 mm); eluent: 60% CH_3_CN/H_2_O; flow rate: 3 mL/min (blue line: UV-254 nm; red line: gamma-ray).


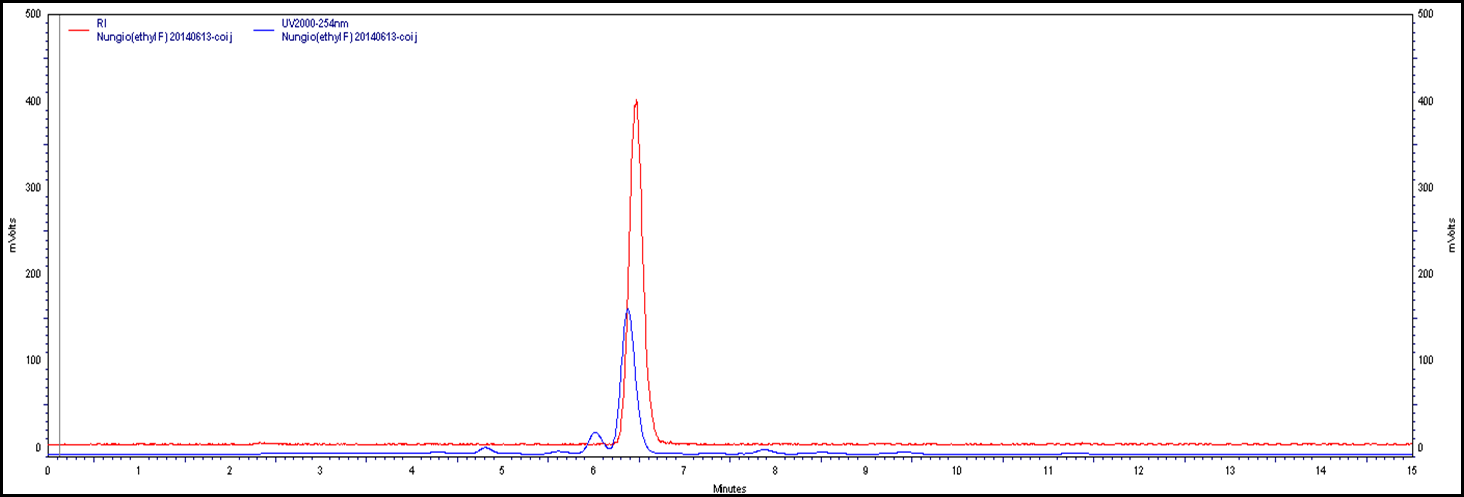


**Fig. S2** HPLC co-injection chromatogram of pure [^18^F]CB251 with an authentic compound **1**; HPLC analytic column (Waters, Xterra RP-18, 5 μm, 4.6 x 250 mm); eluant: 65% CH_3_CN/H_2_O; flow rate: 1 mL/min (blue line: UV-254 nm; red line: gamma-ray).

**II. Stability of [^18^F]CB251 in human serum**

**
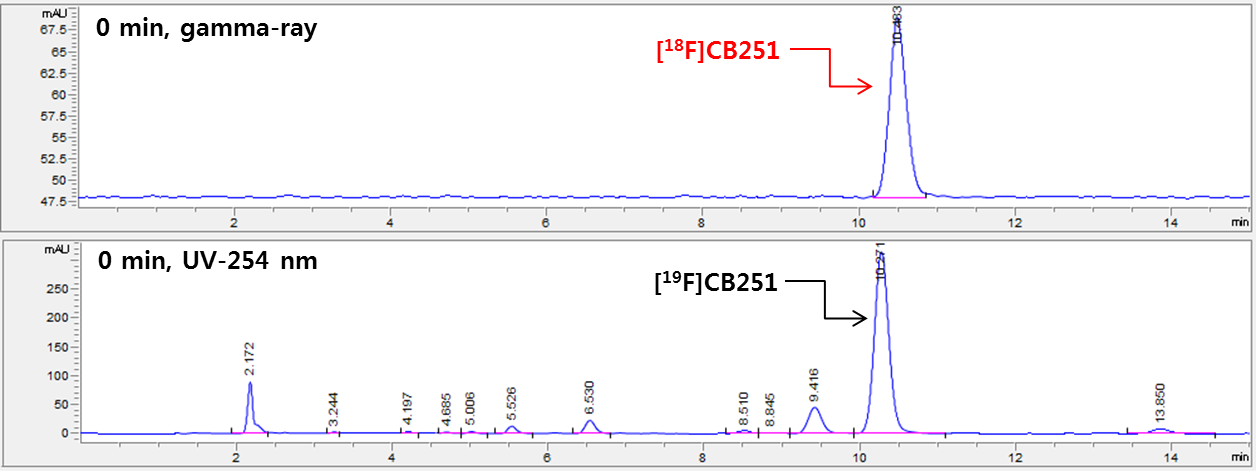
**

**
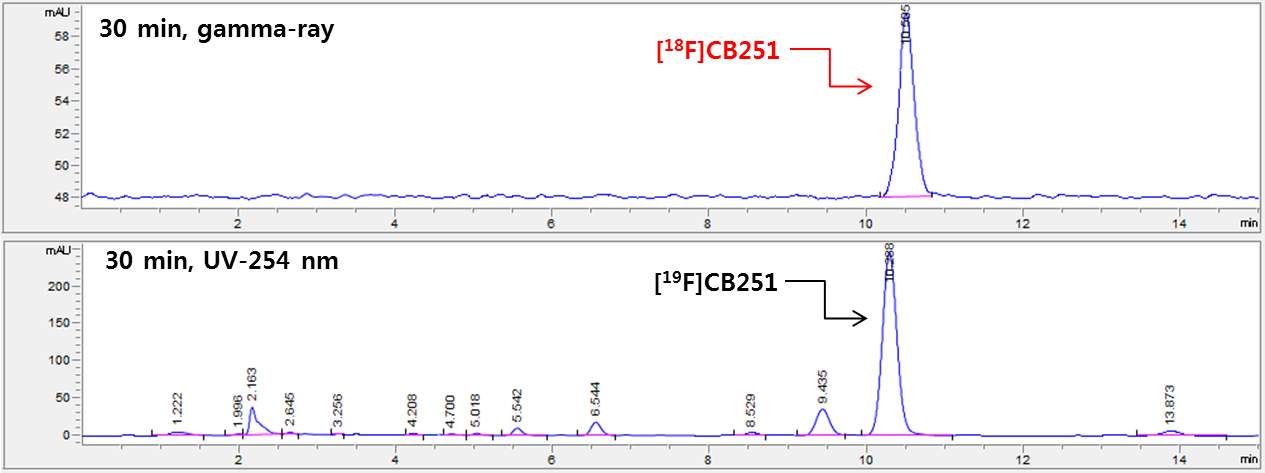
**

**
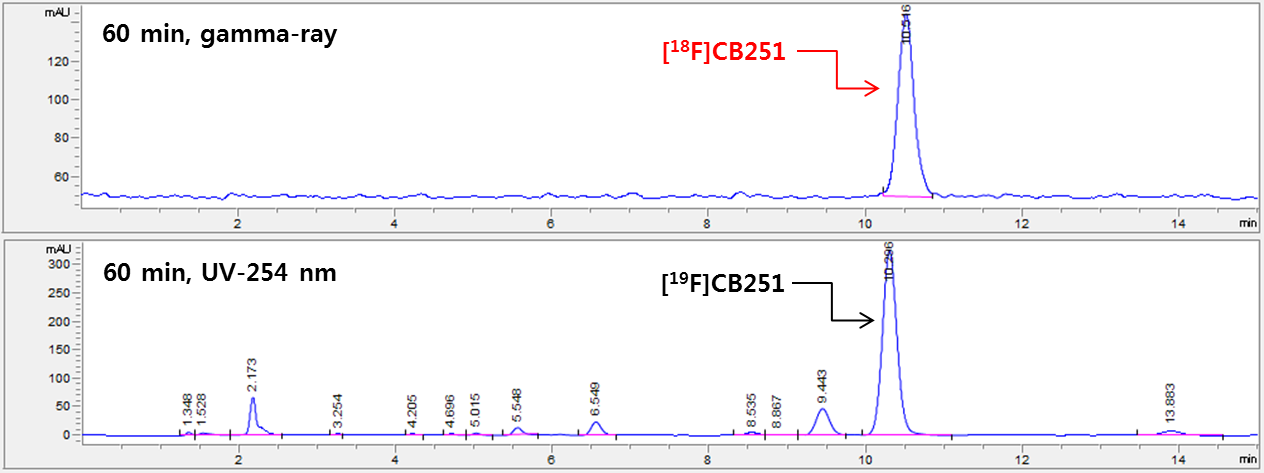
**

**
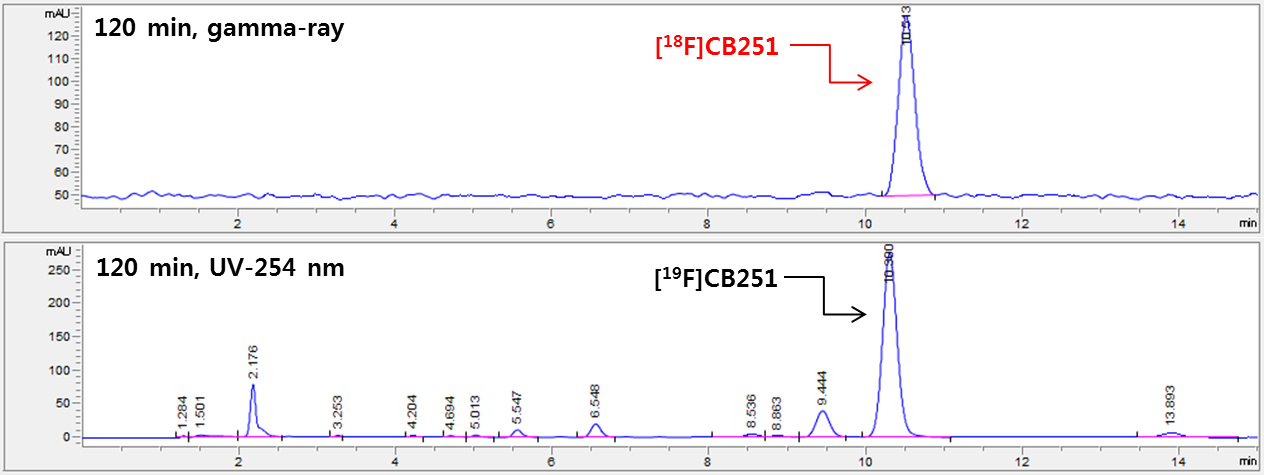
**

**Fig. S3** HPLC co-injection chromatogram of serum samples with an authentic compound **1**; HPLC analytic column (Waters, Xterra RP-18, 5 μm, 4.6 x 250 mm); eluant: 60% CH_3_CN/H_2_O; flow rate: 1 mL/min.
